# Data note on the Avian Contact Study: a questionnaire resource for avian influenza public health planning

**DOI:** 10.12688/wellcomeopenres.23064.1

**Published:** 2024-10-17

**Authors:** Amy C. Thomas, Suzanne Gokool, Genevieve Clapp, Harry Whitlow, Carmel McGrath, Peter Moore, Maud Helwegen, Mariam Logunleko, Irene Bueno, Mick Bailey, Sarah Masterton, Jo Taylor-Egbeyemi, Ian Brown, Richard Puleston, Riinu Pae, Louise E Smith, Sarah Lambton, Ellen Brooks-Pollock

**Affiliations:** 1 Population Health Sciences, University of Bristol Medical School, Bristol, England, UK; 2 The National Institute for Health and Care Research, Health Protection Research Unit in Behavioural Science and Evaluation, Population Health Sciences, University of Bristol Medical School, Bristol, UK; 3 The National Institute for Health and Care Research Applied Research Collaboration West, University Hospitals Bristol and Weston NHS Foundation Trust, Bristol, UK; 4 Faculty of Health and Applied Sciences, University of the West of England School of Health and Social Wellbeing, Bristol, England, UK; 5 University of Bristol School of Veterinary Sciences, Langford, England, UK; 6 UK Health Security Agency, London, UK; 7 Pirbright Institute, Pirbright, England, UK

**Keywords:** Avian influenza, influenza, zoonotic influenza, questionnaire, zoonoses, contact patterns, vaccination, spillover

## Abstract

The Avian Contact Study is a cross-sectional questionnaire of poultry workers and other individuals who have contact with domestic and/or wild birds in the UK. The questionnaire was launched online in May 2024 and in-person responses were gathered at the British Pig and Poultry Fair in Birmingham on 15 and 16 May 2024. This Data Note documents questionnaire development, content and delivery methods. Data collected include information on demographics, seasonal influenza vaccination, avian influenza exposure, contact with birds and people, and awareness of avian influenza. This Data Note provides insights into the first data release collected up to 31 July 2024 for 225 respondents. Data have been released as a University of Bristol held dataset available upon request. The Avian Contact Study provides a pilot resource for research into avian influenza from a zoonotic perspective.

## Introduction

Influenza A virus is a respiratory pathogen causing seasonal epidemics. Sporadic pandemics involve strains derived from animal reservoirs ^1^. In 2021–2022, 48 million birds were culled due to outbreaks of high pathogenicity avian influenza (HPAI) H5N1 across Europe, including many more deaths in wild and domestic birds ^2^. For the first time, transmission of H5N1 in wild birds continued throughout summer in Great Britain and northwest Europe ^3^. Zoonotic influenza A infection in humans is rare and isolated, with symptoms ranging from asymptomatic or mild to severe, and in some cases, fatal. Since 2003, 891 human infections with A(H5N1) viruses, including 463 deaths (CFR 52%) have been reported globally to the World Health Organisation ^4^. Since 2021, there have been 27 global laboratory confirmed cases of A(H5N1) in humans, 5 of which were in the UK ^5^. High numbers of infected birds during the 2021–2022 waves heightened public health concerns and risk assessments ^6^ due to the risk of spillover to humans.

In the UK, current public health policy for A(H5N1) includes local health protection teams responding to HPAI incidents. Individuals who may have been exposed are identified and followed up for close monitoring of symptoms, asymptomatic testing, and possibly antiviral administration ^7^. However, these protocols were formulated during periods of low avian influenza transmission, reducing scalability and efficiency of public health responses under high transmission scenarios. We sought to address data gaps for informing zoonotic avian influenza public health policy through developing and deploying a questionnaire to people who have contact with any type of bird(s) in the UK ( www.bristol.ac.uk/avian-contact).

## Methods

### Eligibility

All UK residents (England, Northern Ireland, Scotland, Wales) aged ≥18 years old and having had contact with domestic and/or wild birds were eligible to participate.

We anticipated the farming community to be a large proportion of our sample. Farmers often work long, unsocial working hours and live in isolated rural communities. From our previous research implementing a questionnaire to cattle farmers on zoonotic diseases ^8^, we found meeting in-person is optimal for research study recruitment and participation versus online. Therefore, the questionnaire was delivered initially in-person by research team members in May 2024 at the British Pig & Poultry Fair, Birmingham, UK ( https://pigandpoultry.org.uk). Following the event, the questionnaire was self-completed online. Data for this data note were collected up until midnight on 31 July 2024. Respondents completing the questionnaire in-person were given a £5 Love2Shop voucher to thank them for their participation.

Questionnaire study data were collected and managed using Research Electronic Data CAPture (REDCap) tools, hosted using a licence at the University of Bristol. REDCAP is a secure, web-based software platform designed to support data capture for research studies ^9^. The final questionnaire (REDCap PDF) used is available with the associated data dictionary as *Extended data* ^10^.

### Content design

Content was created to address the primary need of informing and directing proportionate public health responses to avian influenza during the H5N1 epizootic:

- Inform public health responses to avian influenza through collecting information on motivations behind testing, medication, vaccination behaviours and risk perception
- Understand potential transmission scenarios of HPAI among people who have contact with domestic or wild birds through collecting information on contact patterns for input into disease transmission models

***Questionnaire development***

The questionnaire was developed alongside avian influenza public health teams (UK Health Security Agency; UKHSA) and veterinary government scientists (Animal and Plant Health Agency; APHA).

Published literature searches were carried out to identify questionnaires relevant to this study to inform the development of the structure-based questionnaire. Motivation questions were derived from the FluSurvey ^11^. Social contact questions were adapted from Gimma *et al.* ^12^, POLYMOD ^13^ and Danon *et al.* ^14^. UKHSA investigators assisted with the questions centred around vaccination and medication uptake, testing for avian influenza, bird contact and exposures, and knowledge and risk perception of avian influenza. Occupational job titles as given in the Office for National Statistics Extended Standard Occupational Classification (SOC) 2020 Framework ^15^ were used to guide selection of most relevant occupations for the target demographic. If a respondent’s job title was not listed, a free-text option was available. Free-text options were manually cleaned and consolidated when appropriate.

***Patient and Public Involvement***

Patient and Public Involvement was used via telephone consultations with poultry farmers owning broiler, layer and point of lay farms, for their contribution to improving the design of this study and the questionnaire. The discussions included their description of the types of contacts they have with birds, their motivation for participating in a study of this type and the best method for the delivery of the questionnaire (online/telephone/in-person). Their responses informed the decision for the research team to attend poultry events for in-person recruitment for participation in this study and wording used for the questionnaire, in particular for the question “What type of contact have you had with any type of bird(s) on a typical day?”. Further, a contact with birds was defined following this discussion with public contributors and UKHSA, aligning the definition with the definition used in the UKHSA asymptomatic testing pilot.

To ensure the language used in the Avian Contact Study questionnaire was accessible to all potential respondents, reviews were performed by two members of the plain English panel based at the National Institute for Health and Care Research Applied Research Collaboration West ^16^.

***Questionnaire format***

The questionnaire included 40 questions split across 4 sections and included information on:

Section A: *“About you”*

- Age, gender, postcode of home and work location, main occupation, country of birth, perceived health status
- Knowledge about eligibility to receive a seasonal influenza vaccine in the last 12 months
- Uptake of seasonal influenza vaccine in the last 12 months
- Reasons for and for not receiving a seasonal influenza vaccine
- Knowledge of exposure to avian influenza
- Self-reported avian influenza testing and antiviral medication

Section B: *“Your contact with any type of domestic or wildlife birds”*

- Frequency and type of direct contact with domestic and wild birds where direct contact was defined as being within 2 metres of a bird.
- Biosecurity measures to limit risk of catching avian influenza
- Livestock and domestic pet ownership

Section C: *“Your contact with other people”*

- Frequency and type of contact with people, either relatives, friends, work colleagues and/or others. Direct contact was defined as either:
  ○ Physical: when you met someone face-to-face and had physical contact either by a handshake, hug, kiss or contact sport
  ○ Conversational: you met someone and exchanged at least a few words with the person
  ○ By distance: you were within 2 metres of this person

Section D: *“Your awareness of avian influenza (bird flu)”*

- Knowledge of recent avian influenza outbreaks
- Risk perception of avian influenza to birds and people
- Attitudes towards control measures

### Invitation and reminder strategy

The questionnaire was launched and delivered in-person at the British Pig and Poultry Fair between 15 and 16 May 2024. Following the fair, information for remotely completing the questionnaire online was distributed among contacts met in-person at the fair, to key members of animal health protection teams at the Animal and Plant Health Agency, via poultry farming/keeping forums on social media and contacts at city farms, livestock feed distributors and poultry rehousing networks. These networks publicised the questionnaire.

### Response rate

**Figure 1.**
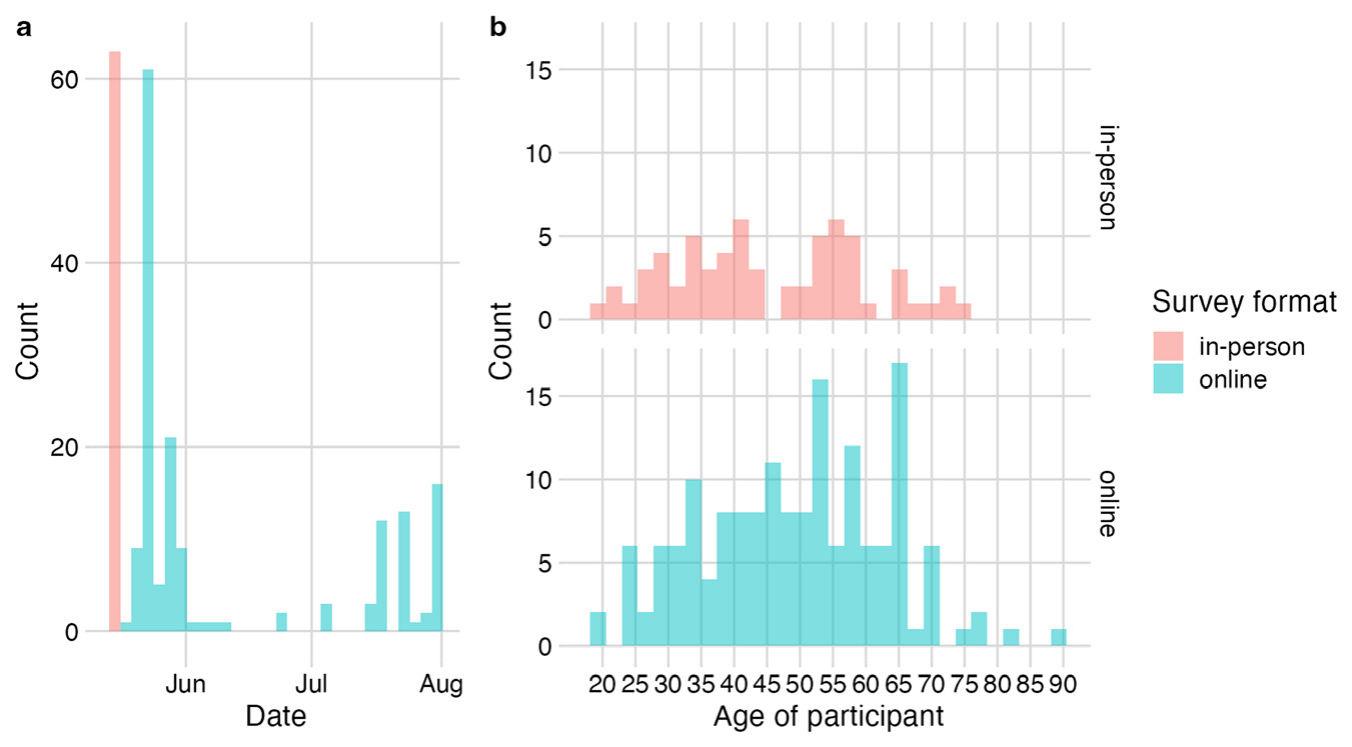
Survey response rates. **a**, Response rate over time by survey format: n=63 in-person (pink) and n=162 online (blue); **b**, age distribution of survey respondents by survey format (in-person/online).

As of 31 July 2024 (data release v1), a total of 225 people had completed the survey, of which 63 were in-person at the Pig & Poultry event and 162 were remotely recruited and self-completed the online questionnaire. There were two spikes in responses in late May, in-line with communications sent out via email to farming and veterinary networks. Responses continued to gradually increase from June following further targeted communications among relevant social media groups, city farms and poultry organisations ( Figure 1a). Most respondents were aged between 30 – 59. For older adults aged ≥65 years, a higher number of responses were collected online compared to inperson ( Figure 1b).

### Key results

***Demographic***

The characteristics of responders according to key variables that will be released with the dataset are shown in Table 1. The median age was 48 years (range 19 – 89) with most respondents identifying as male (63%), poultry farmers (46%) and had worked in their respective occupation for 5 years (median). Most respondents were UK born (92%) with the majority born in England (72%).

**Table 1.** Characteristics of questionnaire respondents. For categorical variables (%) or median (range) for continuous variables.

	N=225
**Age in years**	
Median [Min, Max]	48.0 [19.0, 89.0]
**Age in years**	
19 – 29	21 (9%)
30 – 39	48 (21%)
40 – 49	46 (21%)
50 – 59	60 (27%)
60 – 64	22 (10%)
≥65	28 (12%)
**Gender**	
Female	81 (36%)
Male	142 (63%)
Other	1 (<1%)
Missing	1 (<1%)
**Occupation**	
Poultry farmer	104 (46%)
Veterinarian	16 (7%)
Zookeeper	15 (7%)
Retired	10 (4%)
Farm manager	7 (3%)
Mixed/Livestock/Arable farmer	6 (3%)
Other *	67 (30%)
**Duration in occupation in** **years**	
Median [Min, Max]	5.00 [1.00, 7.00]
**Country of birth**	
England	163 (72%)
Northern Ireland	7 (3%)
Scotland	20 (9%)
Wales	6 (3%)
Not UK born	17 (8%)
Missing	12 (5%)

*Due to small numbers, occupational titles with ≥5 observations per group are shown, otherwise grouped as ‘Other’ here for presentation purposes.

***Health, avian influenza infection, testing and antivirals***

**Figure 2.**
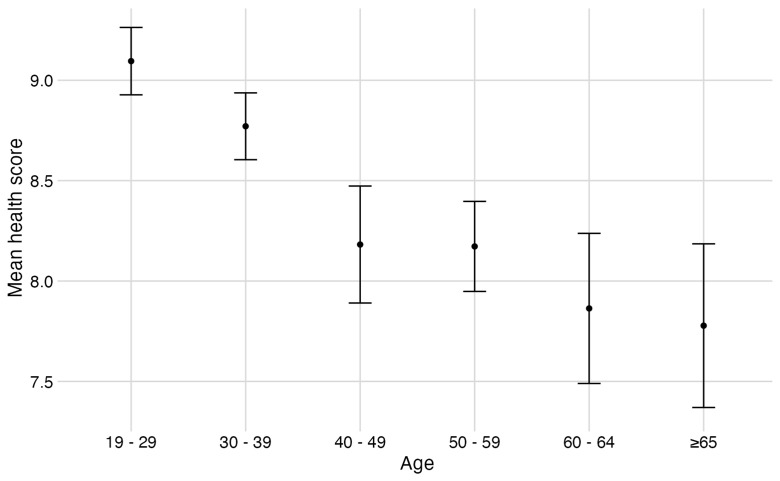
Mean health score by age. Respondents (n=220) rated their overall health on a scale of 1 to 10. Error bars represent standard error of the mean.

Respondents were asked to rate their health on a scale of 1 to 10. Mean health score declined with increasing age ( Figure 2). Thirty-one of 223 respondents reported having been exposed to avian influenza (13%; note 2 respondents did not answer this question). Six people reported being tested previously, of which none were confirmed positive. Antivirals were offered to 23 people, of which 18 accepted.

***Bird ownership and contact with birds***

**Figure 3.**
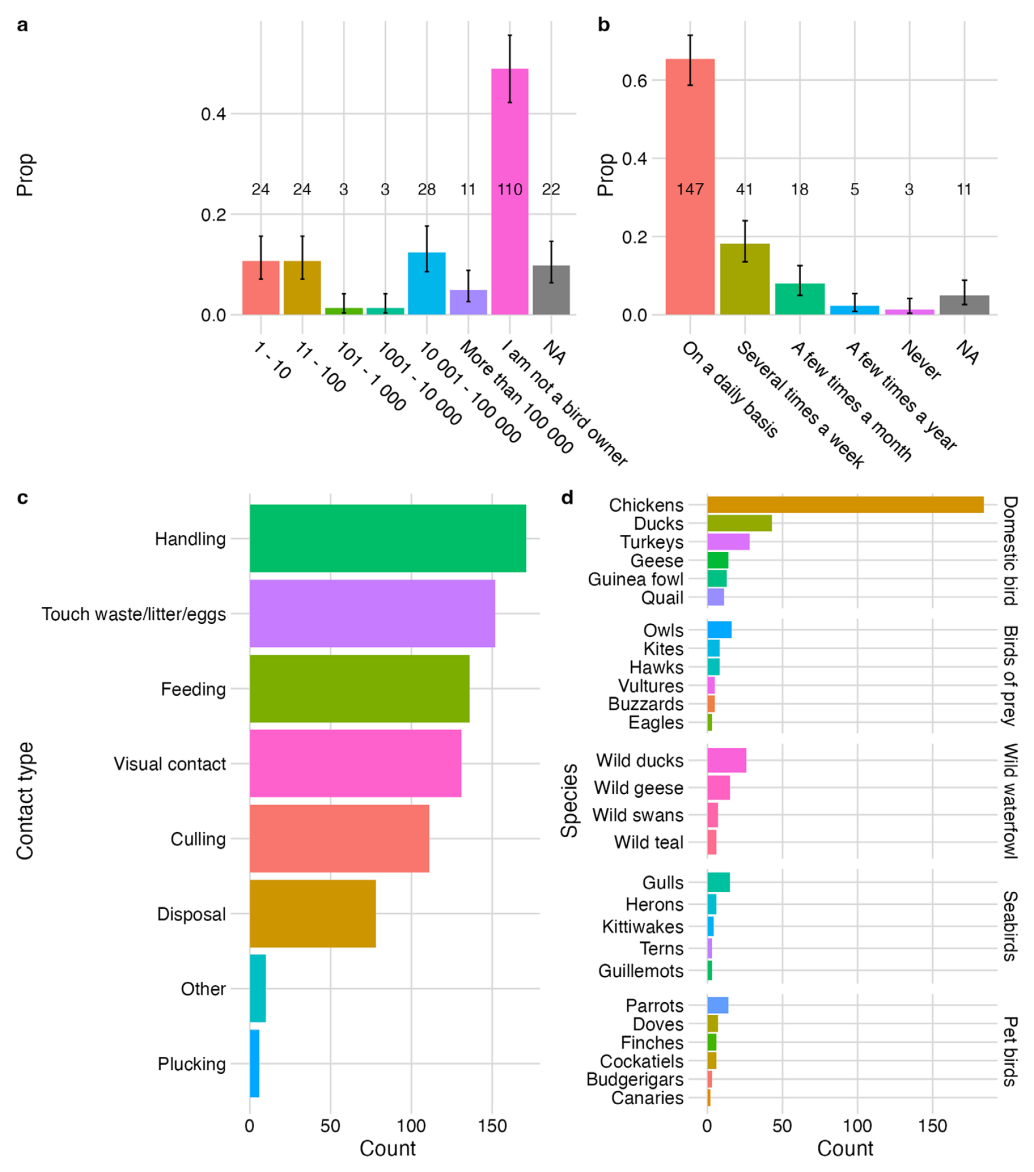
Bird ownership and contact with birds. **a**, proportion and count of respondents owning no birds or birds of various flock sizes; **b**, proportion and count of respondents in contact with birds by contact frequency. **c**, count of types of contact between respondents and birds; **d**, count of reported bird species respondents had contact with. Error bars represent 95% CI for single proportion. NA represents missing values for where respondents did not complete the question.

Of the respondents that reported owning birds (93/203), individuals were either hobby keepers with 1 – 100 birds (12%) or were likely to have large flock sizes of over 10,000 birds (19%). Flock sizes between 101 – 10,000 birds were rarely reported (3%) ( Figure 3a). Respondents frequently reported having daily contact with birds (69%; 147/214) ( Figure 3b). Handling was the most reported type of contact with birds, followed by touching waste/litter/eggs ( Figure 3c). The top 10 most frequently reported species that respondents reported contact with were chickens followed by ducks (both domestic and wild), turkeys, owls, wild geese, gulls, geese, parrots and guinea fowl ( Figure 3d).

### Strengths and limitations of these data

The Avian Contact Study presents a recent snapshot of information between May – July 2024, gathered from individuals in contact with domestic and/or wild birds in the UK, following two seasons of unprecedented transmission of HPAI A(H5N1). The data collected will be used to provide timely descriptions on individuals knowledge, perception of risk and protective behaviours towards avian influenza. Data will also be used to describe the contact patterns of individuals in contact with birds, helping to inform mathematical models of transmission. Currently data on contact patterns for this demographic are very limited ^13^.

Our approach of in-person and remote completion of this online questionnaire widened outreach across different groups, as evidenced by differences in completion across occupational groups – all respondents who were retired or zookeepers completed the questionnaire online. Further, a greater number of older adults aged ≥65 years completed the questionnaire online. A limitation of this approach is that respondents attending the Pig & Poultry Fair are likely to represent a unique cohort with underrepresentation of certain groups because they did not attend or access communication networks used in promotion, e.g. those whose roles don’t allow travel to such events, such as the frontline poultry workforce and smaller scale keepers, or those who don’t speak English as a first language. We note that of those keeping birds, flock sizes between 101–10,000 birds were rarely reported. We do not know how representative our sample is in relation to small/medium/large flock sizes of UK farmers since we could not identify publicly available data for comparison.

We are unable to accurately quantify response rates since individual invitations to complete the questionnaire were not sent out nor were individuals approached in-person recorded. Initial piloting of the questionnaire did not identify missing occupations such as ‘catcher’ or ‘game birds’ as a species group, resulting in free-text responses and potentially missing data. Future iterations should consider these groups. A key limitation is that the sample is skewed towards individuals occupationally exposed to birds, mostly poultry farmers, therefore generalisation to other occupational groups and those with non-occupational contact should be done with caution. Caution should also be taken with sub-group analyses due to small sample sizes. With A(H5N1) having increasing impact on wildlife, future data collection should consider targeted efforts for surveying individuals in this sector. Data are available for researchers as described below.

R version 4.4.0 was used for all analyses and the source code used can be found on Zenodo ^17^.

## Ethics and consent

Ethical approval for the study was obtained from the University of Bristol, Faculty Research Ethics Committee, approval number 17048 on 16 January 2024.

Informed written consent (using e-consent hosted on REDCap) for the use of data collected via the questionnaire was obtained from respondents.

## Figures and Tables

**Table:** 

Author	Contribution
Amy Thomas	Conceptualization, Data Curation, Formal Analysis, Funding Acquisition, Investigation, Methodology, Project Administration, Visualization, Writing – Original Draft Preparation, Writing – Review & Editing
Suzanne Gokool	Investigation, Methodology, Project Administration, Writing – Review & Editing
Genevieve Clapp	Investigation, Project Administration, Writing – Review & Editing
Harry Whitlow	Investigation, Writing – Review & Editing
Carmel McGrath	Methodology, Writing – Review & Editing
Peter Moore	Investigation, Methodology, Writing – Review & Editing
Maud Helwegen	Investigation, Writing – Review & Editing
Mariam Logunleko	Investigation, Writing – Review & Editing
Irene Bueno	Writing – Review & Editing
Mick Bailey	Writing – Review & Editing
Sarah Masterton	Investigation, Writing – Review & Editing
Jo Taylor	Investigation, Writing – Review & Editing
Ian Brown	Writing – Review & Editing
Richard Puleston	Writing – Review & Editing
Riinu Pae	Investigation, Methodology, Writing – Review & Editing
Louise E Smith	Investigation, Methodology, Writing – Review & Editing
Sarah Lambton	Methodology, Writing – Review & Editing
Ellen Brooks-Pollock	Conceptualization, Project Administration, Funding Acquisition, Investigation, Methodology, Supervision, Writing – Review & Editing

## Data Availability

Repository data.bris: The Avian Contact Study: questionnaire data 15 May – 31 July 2024. Due to the sensitivity of the data involved, these data are published as a restricted dataset at the University of Bristol Research Data Repository data.bris, at https://doi.org/10.5523/bris.3nmqsrbv5ruom2abn0ql6e8yh2 ^18^. The metadata record published openly by the repository at this location clearly states how data can be accessed by bona fide researchers. Requests for access will be considered by the University of Bristol Research Data Service, who will assess the motives of potential data re-users before deciding to grant access to the data. No authentic request for access will be refused and re-users will not be charged for any part of this process. This project contains the following underlying data: Data file 1. (Raw underlying questionnaire data – csv file) Data file 2. (Raw underlying questionnaire data - .RDS file) Data file 2. (Associated data dictionary – csv file) Data file 3. (Code for importing underlying data in csv format into R for setting up labelled data - .r file) Data file 4. (Blank consent form and participant information sheet – pdf file) Data are available under the terms of National Archives’ Non-Commercial Government Licence for public sector information. Repository Zenodo: The Avian Contact Study Questionnaire and Data Dictionary [ 10.5281/zenodo.13617061] ^19^ This project contains the following extended data: AvianInfluenzaSocialContactSu.pdf (The final questionnaire REDCap – PDF) AvianInfluenzaSocialContactSur_DataDictionary_080824v1.csv (Associated data dictionary -csv file) Data are available under the terms of the Creative Commons Attribution 4.0 International license (CC-BY 4.0).

## Software availability

Source code available from: https://github.com/amythomas/aviancontactstudy

Archived source code at time of publication: https://zenodo.org/records/13352086 ^17^

License: Creative Commons Attribution 4.0 International license (CC-BY 4.0).
