# Supplementary material for: Data note on the Avian Contact Study: a questionnaire resource for avian influenza public health planning
Source: Wellcome Open Res. 2025 Feb 24;9:604. Originally published 2024 Oct 17. [Version 2] doi: 10.12688/wellcomeopenres.23064.2 (PMC11955075; doi:10.12688/wellcomeopenres.23064.2)
Supplement: Peer review history

# Reviewer report: Reviewer response for version 2

**DOI:** 10.21956/wellcomeopenres.26307.r119360

**Published:** 2025-03-29

**Authors:** Lyle Fearnley

**Affiliations:** 1 Singapore University of Technology and Design, Singapore, Singapore

I am satisfied with the revisions.

Are sufficient details of methods and materials provided to allow replication by others?

Yes

Is the rationale for creating the dataset(s) clearly described?

Partly

Are the datasets clearly presented in a useable and accessible format?

Yes

Are the protocols appropriate and is the work technically sound?

Yes

Reviewer Expertise:

Medical anthropology, human-animal relations, zoonotic diseases

I confirm that I have read this submission and believe that I have an appropriate level of expertise to confirm that it is of an acceptable scientific standard.

# Reviewer report: Reviewer response for version 2

**DOI:** 10.21956/wellcomeopenres.26307.r119359

**Published:** 2025-03-12

**Authors:** Laura Christl Roberts

**Affiliations:** 1 Dept of Agriculture, Western Cape Government, Elsenburg, Western Cape, South Africa; 2 Dept Production Animal Studies, Faculty of Veterinary Science, University of Pretoria, Pretoria, Gauteng, South Africa

The article has been significantly improved. The additions to the introduction provide better background about poultry holdings in England and more information on the distribution of respondents. The extra information on how public contributors and respondents were contacted is also helpful, as is the improved coverage of the different results sections. 

 I am satisfied with the current revised article and have no further comments.

Are sufficient details of methods and materials provided to allow replication by others?

Yes

Is the rationale for creating the dataset(s) clearly described?

Yes

Are the datasets clearly presented in a useable and accessible format?

Partly

Are the protocols appropriate and is the work technically sound?

Partly

Reviewer Expertise:

I work as a government veterinary epidemiologist, involved with avian influenza outbreaks in commercial poultry and in wild birds. My PhD investigated the epidemiology and response to avian influenza in seabirds. I feel competent to assess the article.

I confirm that I have read this submission and believe that I have an appropriate level of expertise to confirm that it is of an acceptable scientific standard.

# Reviewer report: Reviewer response for version 1

**DOI:** 10.21956/wellcomeopenres.25400.r110783

**Published:** 2024-11-26

**Authors:** Lyle Fearnley

**Affiliations:** 1 Singapore University of Technology and Design, Singapore, Singapore

This is a crucial survey to conduct and will be very valuable. We need to know more about the behaviors that are putting people in contact with birds around HPAI risks. My one suggestion is that the analytic significance could be further clarified. What is the hypothesis or underlying research question that will be addressed by the survey? For example, it could be very interesting to compare how contact-related behaviors differ for different occupational roles; or for different species. Yet to do this effectively you might need to specify these questions in the study design in order to develop an appropriate sampling strategy.

Are sufficient details of methods and materials provided to allow replication by others?

Yes

Is the rationale for creating the dataset(s) clearly described?

Partly

Are the datasets clearly presented in a useable and accessible format?

Yes

Are the protocols appropriate and is the work technically sound?

Yes

Reviewer Expertise:

Medical anthropology, human-animal relations, zoonotic diseases

I confirm that I have read this submission and believe that I have an appropriate level of expertise to confirm that it is of an acceptable scientific standard, however I have significant reservations, as outlined above.

# Response

**Published:** 2025-02-18

**Authors:** Amy Thomas

**Affiliations:** University of Bristol, UK

Thank you for your review. We included rationale for developing and deploying the questionnaire under the heading ‘Content design’. In the first para of the ‘Strengths and limitations of these data’ we make some analytical suggestions of what these collected data will be able to investigate, for example, describing contact patterns among those who work with birds to inform mathematical models of transmission.  

 Specific research questions will be addressed in linked papers, therefore we do not think it is necessary to outline specific hypotheses or underlying research questions within the Data Note itself.

# Reviewer report: Reviewer response for version 1

**DOI:** 10.21956/wellcomeopenres.25400.r107397

**Published:** 2024-11-14

**Authors:** Laura Christl Roberts

**Affiliations:** 1 Dept of Agriculture, Western Cape Government, Elsenburg, Western Cape, South Africa; 2 Dept Production Animal Studies, Faculty of Veterinary Science, University of Pretoria, Pretoria, Gauteng, South Africa

**Article summary**

 This data note reports on methodology and results of a questionnaire, administered online and in-person at a poultry fair, aimed at people living in the UK, who have contact with birds. The objective was to fill knowledge gaps in order to inform public health planning for scenarios with large-scale zoonotic transmission of avian influenza viruses. Questions were centred around: demographics and seasonal influenza vaccine awareness and uptake; previous avian influenza exposure, testing and treatment; type and scale of exposure to birds and biosecurity measures to prevent avian influenza spread; type and extent of contact with other people and; knowledge of avian influenza risks. Summaries of respondents' demographics, avian influenza exposure, testing and response and bird ownership and contact were included as "Key Results". 

 The information sought by this study is important for understanding potential repercussions and management options for large-scale zoonotic avian influenza transmission. The representativeness and validity of data obtained through questionnaires is often difficult to assess, as it is in this case, but more effort to describe the target population and calculate useful sample sizes could assist with this.

**Major comments**

**General  comments**

 The purpose of a data note seems to be to provide information to other researchers who may want to use the dataset provided and to include the details about how the data was collected and what the dataset contains, as an indication of how the data can be used and what questions it can answer. 

 Representativeness is a key characteristic that should be described to guide potential dataset users. Given that all bird owners must now register with the UK government (https://www.gov.uk/guidance/register-as-a-keeper-of-less-than-50-poultry-or-other-captive-birds), has an attempt been made to request data on numbers of poultry keepers and the numbers of poultry they own? Similarly, were ideal sample sizes calculated for different groups? This will help demonstrate whether the work was technically sound.

 This dataset does not seem to be available for review, so content, accessibility and format cannot be assessed. 

**Specific Comments**

*Key results, Demographic*:

 One indicator of the representativeness of the data and its potential for re-use would be the spatial distribution of the respondents. Is it possible to indicate e.g. respondents per county, from postcodes provided?

 Results of two of four questionnaire sections are included. I suggest that some information about contact with other people and avian influenza awareness is added.

**Minor Comments**

**Introduction**

 Paragraph 1:

- Specify that the “seasonal epidemics” are in humans
- Second sentence: reword “including” wild birds to “in addition to” if that is correct, and rephrase so that the sentence does not imply that more wild/ backyard birds died than commercial poultry.
- Clarify that the human cases mentioned since 2021 have been largely the recent clade 2.3.4.4b viruses and that there has been no evidence of human-to-human transmission.
- 5 UK cases- Two cases at least do not seem to have been true infections but rather just contamination. Please double check: https://www.gov.uk/government/publications/avian-influenza-influenza-a-h5n1-technical-briefings

Paragraph 2:  clarify that the “high transmission scenarios” have not yet been experienced but that they require contingency plans.

**Methods**

*Eligibility*, paragraph 2:

- Rephrase: “We anticipated the farming community to be a large proportion of our sample” – it sounds like you expected it would be large proportion, rather than that you needed representation of an important group. Maybe say “we needed to ensure the farming community was represented but... unsocial hours etc"
- Provide a short description of the pig and poultry fair and the people who generally attend.
- At what point in the questionnaire were people without bird contact excluded?

*Content design, Questionnaire developmen*t: should “structure-based questionnaire” be a “structured questionnaire”?

*Content design, Patient and public involvement*

- I see that Wellcome Open Research suggests this section is included, but since there were no patients involved, rather just call the section “Public Involvement” and say that, “we involved poultry farmers in questionnaire design via telephone consultations..” (I’m not sure public involvement can be “used”).
- How many poultry farmers were consulted on questionnaire design and how were they chosen?

*Invitation and Reminder Strategy: *“Poultry rehousing” is not a term that I’m familiar with- please check it is correct and if it is, provide a short explanation.

**Key results**

*Demographic*:

- The age of people responding online vs in-person does not seem relevant and could be considered analysis, which is not required by the Wellcome Open Research Date Note guidelines. Suggest excluding the related text and figures.
- How will country of birth affect any of the variable that could be examined? I suggest it is excluded from the description of results. Respondents who moved to the UK after average working age could be more relevant.
- The description of decreasing health with age is a result of analysis. Suggest it is excluded
- Were the people who were offered antivirals all exposed to AIV? This would depend on the public health policy in the UK and is worth clarifying.

*Bird ownership and contact, Figure 3*

 The data in figure 3 would be better presented as tables. Given that sampling was not planned to be representative, the proportions shown by the charts are less relevant that the numbers of respondents that fitted into each category and potential sample sizes to be used for further study. 

**Ethics and Consent**

 Please add the "informed written consent" section to the questionnaire, if there was more information provided to respondents. It's important to demonstrate that respondents were assured that their data would be kept confidential, with no way of identifying them. Poultry farmers in particular may not answer or answer differently if they thought the government had any way to use there answers against them.

Are sufficient details of methods and materials provided to allow replication by others?

Yes

Is the rationale for creating the dataset(s) clearly described?

Yes

Are the datasets clearly presented in a useable and accessible format?

Partly

Are the protocols appropriate and is the work technically sound?

Partly

Reviewer Expertise:

I work as a government veterinary epidemiologist, involved with avian influenza outbreaks in commercial poultry and in wild birds. My PhD investigated the epidemiology and response to avian influenza in seabirds. I feel competent to assess the article but felt that I could not assess the value of the dataset without seeing it or having it slightly better described.

I confirm that I have read this submission and believe that I have an appropriate level of expertise to confirm that it is of an acceptable scientific standard, however I have significant reservations, as outlined above.

# Response

**Published:** 2025-02-18

**Authors:** Amy Thomas

**Affiliations:** University of Bristol, UK

Thank you for your positive and considered review. We have revised the Data Note following the comments and provide our response below.

**General comments** We agree that representativeness is a key characteristic. The UK legislation that all bird owners must now register flocks of any size was opened on 12 December 2023 ( https://www.gov.uk/guidance/register-as-a-keeper-of-less-than-50-poultry-or-other-captive-birds#full-publication-update-history) with an initial deadline to register by 1 October 2024. Even though registration is open-ended, this is a fairly new requirement and unlikely data are accessible until the next annual livestock census (which would likely be Spring 2026 for the majority of records). Furthermore, given most respondents had contact with flocks of >50 birds, it is unlikely these data would significantly contribute to assessing representation for this tranche of data.   However, we have provided additional information in a new paragraph in the introduction using available national data for context describing poultry holdings in England, HPAI epidemiology with regard to numbers of infected premises and detail on the agricultural workforce likely in contact at infected premises. Further, we have plotted the spatial distribution of respondents using supplied postcode data (Figure 2) and interpreted alongside livestock demographic data for poultry in Great Britain, available from APHA ( https://assets.publishing.service.gov.uk/media/669e3a76fc8e12ac3edb011e/lddg-pop-report-avian23.pdf).    As this was a pilot study aiming to scope messaging, recruitment and questionnaire completion, ideal sample sizes were not calculated. However, this pilot can now provide data for informing future, large scale studies.

 Thank you for raising that the dataset was not available for immediate review. The dataset is now Open Access and we have updated the information regarding access.

**Specific Comments** To preserve respondent anonymity in the open access dataset, postcode is not given; however, we have plotted the spatial distribution (see earlier general comment re representativeness).   Thank you for the suggestion to include further information across all questionnaire sections. See results headings ‘Contact with other people’ and ‘Biosecurity measures and awareness of avian influenza’. We have included some information on biosecurity measures alongside ‘Awareness of influenza’ as we feel this information is complimentary and strengthens this section.

**Minor comments, paragraph 1**

1. The term ‘epidemic’ together with the sentence that proceeds it implies these seasonal epidemics related to humans. Epizootic and panzootic would be appropriate, analogous terms for animal populations. Therefore, we’d rather keep the first sentence in para 1 as it is.  
2. Thank you, we rephased ‘including’ to ‘in addition to’.
3. We have clarified that the human cases mentioned since 2021 have largely been the recent 2.3.4.4b clade.
4. The number of five UK cases is originally cited from the World Health Organisation reference ( https://www.who.int/publications/m/item/cumulative-number-of-confirmed-human-cases-for-avian-influenza-a(h5n1)-reported-to-who--2003-2024-26-february-2024). Whilst these five WHO ‘cases’ corroborates with the five ‘detections’ in the UKHSA reference (technical briefing 5) - one detection in 2021 and four detections in 2023, we agree that there are uncertainties in the confirmation of any of these cases being true infections. Table 1 in the UKHSA Technical Briefing 5 gives the likely significance of the detections; two of the 2023 cases were detections of uncertain significance. We have amended this section of the introduction to reflect the uncertainty in cases being true infections.

   
**Minor comments, paragraph 2**

1. This clarification of information regarding not yet experiencing ‘high transmission scenarios’ has been added.

**Minor comments, methods, paragraph 2**

1. “We anticipated the farming community to be a large proportion of our sample”. This has been rephased for clarity that we expected the farming community to represent a large proportion of the sample but owing to their work patterns can have reduced representation.
2. A short description of the P&P Fair has been added.
3. At the pre-screening questionnaire stage people without bird contact were excluded. We have added information on this for clarification.

 
**Methods, ‘Content design, Questionnaire development’**

1. “should “structure-based questionnaire” be a “structured questionnaire?” Yes, amended.
2. The NIHR definition of public involvement does include patients, but we agree that for brevity that Public Involvement alone as a heading is fine; this is representative of our method.
3. We have included additional detail describing how poultry farmers were identified and contacted. Briefly, we consulted four poultry farmers to help shape the study and questionnaire design. Initially, pre-existing links to poultry farmers within the research team were used, as well as internet searches for local poultry farmers. The process was iterative, where outcomes from contributor meetings informed subsequent discussion topics and contributors suggested new contacts within their own networks. We have expanded on this detail in the Public Involvement section and included details on how public contributors were renumerated for their time. 

  “Poultry farmers were identified and contacted initially through existing collaborations of the Avian Contact Study team e.g., through livestock veterinarians and commercial units previously involved with research. Internet searches further identified local farmers who were contacted by email/phone by a researcher. In-depth telephone interviews were conducted with four poultry farmers. This public involvement strategy was an iterative process, with public contributor meetings refining subsequent discussion topics and contributors themselves suggesting further contacts in their network for consultation. Public contributors shaped the wording and focus of the questionnaire and subsequently tested an early version”.   “Public contributors were given a £25 Love2Shop voucher to thank them for their participation.”    

1. Poultry rehousing is a commonly used term in the UK which refers typically to the rehousing or rehoming of poultry from commercial production systems, usually to backyard type systems.

**Key results**

1. We would like to retain these data regarding the age of people responding online vs in-person as they provide a description of respondents obtained both at the Pig & Poultry Fair and subsequently, remotely, online. Although not a Wellcome Open Data Note requirement, this section on the demographic is useful to provide context to potential future data users.
2. In the UK, the frontline commercial poultry workforce is typically non-UK born. This variable may be useful to those who are interested in exploring practices from this cohort/assessing representation. This variable gives context to the questionnaire respondents. We did not collect information on how long a respondent has been living in the UK in relation to their occupation.
3. We would like to retain Figure 2 showing the distribution of mean health score with age since it provides validation of the data i.e., that the relationship shown is expected.
4. Yes, the people who were offered antivirals reported being exposed to AIV. This section details that 31/223 respondents reported being exposed to AIV. Antivirals were offered to 23 people, of which 18 accepted.
5. Figure 3 shows both the number of respondents in each category as well as the proportion. We disagree that a table would be better, the figure gives a visual overview as well as providing counts. The underlying data can be accessed in full from the dataset.

**Ethics and consent**

1. Details of ethics and consent are given under the ‘Ethics and Consent’ heading. “Informed written consent (using e-consent hosted on REDCap) for the use of data collected via the questionnaire was obtained from respondents.”
  The underlying data accompanying the Data Note contains a copy of the blank consent form and participant information sheet. We apologise these could not be accessed readily previously, these are now available Open Access for full details and we have signposted to them in the ‘Ethics and Consent’ heading.
